# Evaluation of Marbofloxacin in Beagle Dogs After Oral Dosing: Preclinical Safety Evaluation and Comparative Pharmacokinetics of Two Different Tablets

**DOI:** 10.3389/fphar.2018.00306

**Published:** 2018-04-10

**Authors:** Zhixin Lei, Qianying Liu, Bing Yang, Haseeb Khaliq, Saeed Ahmed, Bowen Fan, Jiyue Cao, Qigai He

**Affiliations:** ^1^State Key Laboratory of Agriculture Microbiology, College of Veterinary Medicine, Huazhong Agriculture University, Wuhan, China; ^2^Department of Veterinary Pharmacology, College of Veterinary Medicine, Huazhong Agricultural University, Wuhan, China; ^3^National Reference Laboratory of Veterinary Drug Residues and MAO Key Laboratory for Detection of Veterinary Drug Residues, Huazhong Agriculture University, Wuhan, China

**Keywords:** fluoroquinolones, marbofloxacin, pharmacokinetics, Beagle dogs, bioavailability, toxicity

## Abstract

The current study evaluates a tested marbofloxacin tablet (MBT) (Petsen), in terms of bioavailability and pharmacokinetics (PK) in a comparison of the commercialized and standard tablet (Marbocyl) in beagle dogs. Four different bacterial species were selected for the determination of the minimal inhibitory concentration (MIC) against marbofloxacin (MBF). Target animal safety studies were conducted with a wide spectrum of dosages of Petsen. Pharmacokinetics and bioavailability of Petsen were observed after the oral administration of a recommended dosage of 2 mg/kg. The MIC_90_ of MBF against *Staphylococcus aureus, Escherichia coli, Pasteurella multocida*, and *Streptococcus* were 2.00, 4.00, 0.25, and 0.50 μg/ml, respectively. These results showed that the MBT has an expected antimicrobial activity *in vitro*. The main parameters of *t*_1/2β_, Cl_b_, AUC_0−∞_, *C*_max_, and *K*_*e*_ were 22.14 h, 0.15 L/h, 13.27 μg.h/ml, 0.95 μg/ml, 0.09 h^−1^, and 16.47 h, 0.14 L/h, 14.10 μg.h/ml, 0.97 μg/ml, 0.11 h^−1^ after the orally administrated Petsen and Marbocyl, while no biologically significant changes and toxicological significance have been found by their comparison. These findings indicate that the Petsen had a slow elimination, high bioavailability and kinetically similar to the commercialized Marbocyl. Furthermore, no statistically significant differences were distinguished on the continuous gradient dosages of 2, 6, and 10 mg/kg in the term of the clinical presentation. The present study results displayed that the tested MBT (Petsen) was safe, with limited toxicity, which was similar to the commercialized tablet (Marbocyl), could provide an alternative MBT as a veterinary medicine in beagle dogs.

## Background

Marbofloxacin (MBF), belongs to the third-generation synthetic fluoroquinolone antibiotic formulated especially for the veterinary field. Due to its wide range of bactericidal activity, MBF is mostly used against Mycoplasma, Gram-negative, and some of the Gram-positive pathogens (Sidhu et al., 2011; Tohamy and El-Gendy, 2013). It is administered orally or parenterally for the treatment of gastrointestinal and respiratory diseases in pigs and cattle, and has a high bioavailability, near to 100% (Committee for Veterinary Medicinal Products, 2009a; Ding et al., 2010; Tohamy and El-Gendy, 2013; Shan et al., 2014). Due to its broad spectrum it is efficient against canine pathogenic bacteria such as: *Streptococcus* spp., *Proteus* spp., *Staphylococcus* spp., and *Escherichia coli*, and is permitted for the treatment of pet animals at a dosage level of 2.0 mg/kg body weight (b.w.) once a daily, by an oral administration (Soussy et al., 1989; Unmack, 1990; Spreng et al., 1995; Thomas et al., 1997; Paradis et al., 2001).

In a 13-week repeat-dose study with an oral dosage of 1, 4, and 40 mg/kg b.w. MBF in adult dogs, testicular tubular atrophy was observed in only one of the dogs given a dose of 40 mg/kg b.w.; no effects were observed at doses of below than 40 mg/kg. These findings propose that MBF has a low toxicity and a broad dose range (Committee for Veterinary Medicinal Products, 2009b). Additionally, adverse reactions are rarely described in veterinary clinical trials in which MBF has been evaluated (Cotard et al., 1995; Carlotti et al., 1998, 1999; Frazier et al., 2000). Further, MBF has been demonstrated to be a safe for the use in dogs even if used at three times the recommended dose, continuously for 3 months (Bousquetmelou et al., 1997). Inappetence decreased activity, and vomiting were the most commonly observed mild signs. However, there are no available studies of intensive doses from 4 to 40 mg/kg b.w., and a few safety evaluations regarding the toxicity in dogs administered marbofloxacin tablet (MBT).

The pharmacokinetics (PK) actions of MBF have been studied in various animals such as cows, goats, sheep, pigs, cats, and dogs (Waxman et al., 2001; Schneider et al., 2004; Albarellos et al., 2005; Ding et al., 2010; Sidhu et al., 2010a,b, 2011); these studies showed that MBF was widely and rapidly distributed in tissue, with a high bio-distribution in the peripheral tissue and plasma, and showing nearly 100% bioavailability. However, few studies on the PK of MBF in dogs were studied and the previous reports have revealed that MBF has the PK features such as good absorption after oral/parenteral supplementation, higher amounts in tissue than plasma, and weak binding to the plasmatic proteins (<10%) (Schneider et al., 1996; Haritova et al., 2006; Andraud et al., 2011; Sun et al., 2015). MBF is widely distributed throughout the animal's body, which can result in 1.6 times more drug concentration in skin comparing to the plasma of dogs. Moreover, MBF plasma concentrations can sustain above the minimal inhibitory concentration (MIC) (>24 h) longer than the dose density (Schneider et al., 1996). As MBT is a new formulation for treatment in pets, few PK properties are available in previous reports. In the previous plasma PK study, 1.25 and 2.5 mg MBTs (Marbocyl) were performed in beagle dogs (MBT, FDA, Marbocyl), but the recommended dosage (2 mg/kg) by EMA was used in this study. Compared with the Marbocyl, the MBT (Petsen) in this study was compared the toxicity and PK data like bioequivalence in dogs.

Bioequivalence studies support complementary applications in the formulation, route of administration, or manufacturing process that may affect bioavailability (Ozdemir and Yildirim, 2006; Zaid et al., 2017). The criteria for bioequivalence are formulated by their respective organizations, and there are relevant guidelines from regulatory bodies in Europe and the United States. According to guidelines two products are tested in order to prove that active ingredients are available at the site of drug action, following similar assimilation rate and extent (Listed, 1998; Rockville, 2000; Chen et al., 2001; U. S. Food and Drug Administration, 2003; Davit et al., 2016). The similarity is defined by acceptable limits of differences between the pharmacokinetic parameters of compared products (Alp, 2009; Galgatte, 2014; Kaushal et al., 2016). In other words, demonstrating bioequivalence between two drug products means that the same rate and extent of absorption of the active components is assured. Mathematical characterization is: “A tested drug T is bioequivalent with a reference drug R if the 90% confidence interval (CI) for ratios of means μ of main pharmacokinetic parameters - area under the curve (AUC) and maximum concentrations *C*_max_ are included in the 0.8–1.25 interval” (Gherghiceanu et al., 2016). In fact the quantitative, statistical rule implies that even products with different half time of adsorption and halftime of elimination can be bioequivalent.

In this study, we wished to compare Petsen, a product in development which contains MBF, with Marbocyl, which is a marketed veterinary product, in China, containing MBF. Petsen has been developed for veterinary use in treating cats and dogs: both are presented as tablets with 20 mg MBF. Moreover, the drug content and key excipients were similar to the referenced MBT (Marbocyl). In this study, the aim was to assess the evaluation of MBF in beagle dogs after oral dosing, including the preclinical safety evaluation and comparative PK of two different tablets the tested Petsen and the reference formulation Marbocyl. The findings of this study could provide an alternative product for use of MBT in veterinary medicine.

## Materials and methods

### Chemicals and bacteria

The standard substance MBF (100.3% purity, NO. 201104005) was formulated and supplied by Wuhan Huishen Biotechnology Co., Ltd., The MBT (Petsen) (20 mg/tablet; NO.20110505) containing 71.4% MBF per tablet, were formed and supplied by Wuhan Longyu Biotechnology Co., Ltd. The all of the chemical agents used for this analysis were of high-performance liquid chromatography (HPLC) grade, and other organic solvents were of analytical grade. Marbocyl (20 mg/tablet) provided by the French company Vetoquionol S.A. The standard substance MBF was prepared under sterile conditions by addition of a 2% sterilized aqueous solution of acetic acid into a sterile injectable solution, with the concentration of 20 mg/mL by the authors in this study. To test the susceptibility of bacteria to MBF, each isolate was sub-cultured at least three times in tryptic soy broth (TSB) and tryptic soy agar (TSA; Qingdao Hai Bo Biological Technology Co., Ltd., Shangdong, China) containing 5% newborn calf serum (Zhejiang Tianhang Biotechnology Co., Ltd., Zhejiang, China).

### Bacteria strain isolation

Four kinds of bacteria (*Staphylococcus aureus, E. coli, Pasteurella multocida*, and *Streptococcus*) with 50 isolates were selected to determine the MIC of MBF. Each kind of bacteria including 50 strains was isolated from beagle dogs from various Chinese provinces including Hubei, Anhui, Jiangxi, Guangzhou, and Sichuan between 2016 to 2017 years. *E. coli* ATCC 25922 strain was selected to be used as a reference isolate for antibiotic susceptibility determination. Before testing the MIC, each of isolate was sub-cultured at least three times in TSB or TSA.

### Animals

Thirty-six healthy male and female beagle dogs, weighing between 8.0 and 10.0 kg, were selected from the Center of Laboratory Animals of Hubei Province (Wuhan, PR China) and were prepared for PK studies. The animals (dogs) were housed separately in cages under a 12 h light/dark cycle and were offered *ad lib* food and water during this experiment.

Animals did not receive any antimicrobial treatment for 14 days before the experiments. These animals were deemed to be normal and clinically healthy after having a regular body checkup and were thus used for this experiment (Lei et al., 2017c).

The study was ratified by Ethical Committee of Huazhong Agricultural University, Faculty of Veterinary Medicine. All the experiments involving animals were accompanied in accordance with the Guide for the Care and Use of Laboratory Animals of Hubei Provincial Laboratory Animal Public Service Center (permit number SYXK 2013-0044).

### Antimicrobial susceptibility testing

Determination of MBF susceptibility against the four kinds of bacteria was executed using agar dilution technique, according to Clinical and Laboratory Standards (CLSI) guidelines in the previously described study (Lei et al., 2017b,c). Strains (2–4 μl, about 10^8^ CFU/ml) were administrated onto TSA agar plates having newborn calf serum, with two-fold serial dilutions of MBF (0.0625–32 μg/ml). When the MIC values of isolates were over 32 μg/ml, the MBF concentrations in TSA were expanded for detecting. Strain plates were incubated at 37°C for 48 h. MICs were identified as the lowest concentrations of drug that caused the growth inhibition. The MIC-value of *E. coli* (ATCC 25922) to chloramphenicol was used to verify the results of the susceptibility testing.

## Target animal safety evaluation

### Experimental design

Twenty-four beagle dogs (50% males) weighing 8–10 kg and aged 12–14 months old, were selected for inclusion in this study. Dogs were randomly assigned to four groups according to Petsen dose administration. According to the Guiding Principles of Veterinary Drug Research and Development Technology of China, and the Food and Drug Administration (FDA) (Kux, 2011; Shuren, 2012), the dogs in each group were orally administered 0, 2, 6, or 10 mg/kg of Petsen daily, respectively, for 40 continuous days. All dogs in each group were anesthetized with pentobarbital sodium and euthanized at 22–24 h after their last dose. Dogs in the control and high dose groups were selected to investigate the change in visceral organs after day 40. This study complied with the Technical Guidelines of Veterinary Drug research and Good Laboratory Practice Regulations of China (Good Laboratory Practice Regulations of China, 2012).

### Clinical observations

Throughout the study, we observed Beagle dogs at least two-times/day to determine the mortality, morbidity, severity, and duration of any behavior change, evidence of toxicity, as well as to observe the general appearance and abnormalities. Detailed animal health examinations, including temperature, body weight, and food consumption were performed on each animal on day 0, 14, and at the termination of the study (day 40).

### Hematology analysis

Blood and urine were collected from all dogs in each group, for hematological and urine analysis, which was by use of a Coulter HmX Hematology Analyzer (Beckman Coulter Inc., Fullerton, CA, USA) and a UA-66 Urine Analyzer (Shanghai TianChen Technology Inc., China). Blood and urine were collected on day 0, 14, and 40. Hematological evaluations included hemoglobin concentration (HGB), red blood cell count (RBC), white blood cell count (WBC), hematocrit (HCT), and blood platelet count (PLT). Urine analysis included ketone bodies (KET), glucose (GLU), pH, bilirubin (BIL), urobilin (URO), occult blood (BLD), protein (PRO), nitrite (NIT).

### Serum biochemistry

Serum biochemistry was performed with a Synchron Clinical System CX4 (Beckman Coulter, Brea, CA USA) under the manufacturer's guidelines (Beijing Leadman Biochemistry Technology Co. Ltd., Bejing, China). The serum biochemistry evaluations included aspartate aminotransferase (AST), alanine aminotransferase (ALT), alkaline phosphatase (ALP), albumin (ALB), total protein (TP), glutamate (GLU), cholesterol (CHOL), blood urea nitrogen (BUN), creatinine (CREA), triglyceride (TG), creatine kinase (CK), total bilirubin (TBIL), potassium (K), sodium (Na), chloride (CI), inorganic phosphorus (P), and calcium (Ca).

### Histopathological examinations

The main organs of each animal, including heart, liver, spleen, lungs, and kidneys, were weighed separately. Organ weight/100 g body weight was determined on the basis of fasted animal's body weight. The tissues from these organs were kept in 10% neutral buffered formalin until testing. Histopathological study was conducted with routine paraffin-embedding method and sections of 5 μm thickness stained with hematoxylin-eosin were observed with light microscopy to evaluate morphology.

### Pharmacokinetics and bioequivalence study

#### Experimental design

A crossover design was used. Twelve beagle dogs were divided into two groups, with half males and females in each group. One group received a single oral administration of Marbocyl, while the other group received oral administration of Petsen by gavage; the dosage for both groups was 2 mg/kg. After a 14-day washout period, dogs in the two groups were given the alternate treatment, either Marbocyl or Petsen at 2 mg/kg. After another 14-day washout period, 6 beagle dogs were selected from these 12 and given a single i.v. injection of an aqueous solution of the base form of MBF at the same dose. Blood samples were collected at predetermined times as follows: 0, 10, 30, and 45 min, 1, 1.5, 2, 2.5, 3, 4, 6, 8, 12, 16, 24, 36, 48, 60, and 72 h following the administration of the three drug formulations.

Blood samples (2.0 mL) were collected by injecting a 7-gage needle into the forelimb cephalic vein or the hind leg saphenous vein and letting the blood drop into a 5 mL heparinized centrifuge tube. Samples obtained were centrifuged at 3,000 rpm/min for about 15 min. The plasma was immediately removed and stored at −20°C until analyzed.

#### Plasma treatment and HPLC conditions

Plasma samples were thawed to room temperature and MBF in the plasma was extracted. A 0.2 mL plasma sample was placed into a 5 mL polypropylene centrifuge tube; 2 mL chloroform extractant was added. This blend was horizontally vortexed for 5 min and later centrifuged for 6 min at 12,000 rpm. The separated lower layer was shifted into 10 mL centrifuge tube and desiccated at 45°C under a nitrogen stream. The residue was re-dissolved in 200 μL of a 2% aqueous solution of acetic acid. This aqueous solution was centrifuged for 5 min at 5,000 rpm and the supernatant was collected to be analyzed.

Agilent 1100 series equipment was used as the HPLC system, with the variable wavelength indicator (Agilent 1100, G1314-60086). The MBF drug detection was performed at 295 nm using an ultraviolet detector. An automatic injection of 25 μL was measured on an Agilent ZORBAX Extend-C18 stainless steel column (250 × 4.6 mm, 5 μm). The mobile phase was acetonitrile (A) and 1% formic acid aqueous solution (B) (75:25, v/v) with a flow rate of 1.0 mL/min.

#### HPLC method validation

This method was confirmed for plasma, and a standard calibration curve was prepared with plasma concentrations of 5, 0.5, 0.05, and 0.02 μg/mL. Linearity was determined by the standard curve and the precision, accuracy, and recovery were calculated between the standard substance and treated MBF in plasma. The lower limit of detection (LLOD) and the lower limit of quantitation (LLOQ) of MBF were defined as the drug concentrations ensuing in a peak height of three-times, and ten-times, the signal noise, respectively.

#### Pharmacokinetic and bioequivalence analysis

The pharmacokinetic examination was executed by WinNonlin software. Theoretical curves and experimental data were plotted semi-logarithmical and examined with the naked eye. Selection of the best model was performed using Akaike's Information Criterion (Sandulovici et al., 2009). PK parameters were determined for each individual animal, and the routes of administration were compared.

## Statistical analysis

Analysis of variance (ANOVA) was applied to compare the pharmacokinetic parameters of the formulations on the test preparation with the reference ones (Pfaller et al., 2010; Government of Canada HC, 2014). *T*_max_ association was achieved with a Wilcoxon signed rank test. Parametric 90% CIs of the mean of test/reference ratios of AUC_0−∞_ and *C*_max_ were calculated using the residual variance of ANOVA with the assumption of a multiplicative model. Confidence intervals were measured by SPSS analysis (IBM, USA).

MIC_90_ was calculated using SPSS software, and statistical analyzes were performed using Student's *t*-tests, for between-group comparisons of the parameters. The *p* < 0.05 was considered to indicate a statistically significant difference.

## Results

### MIC distributions of the four kinds of bacteria

**Figure 1:**
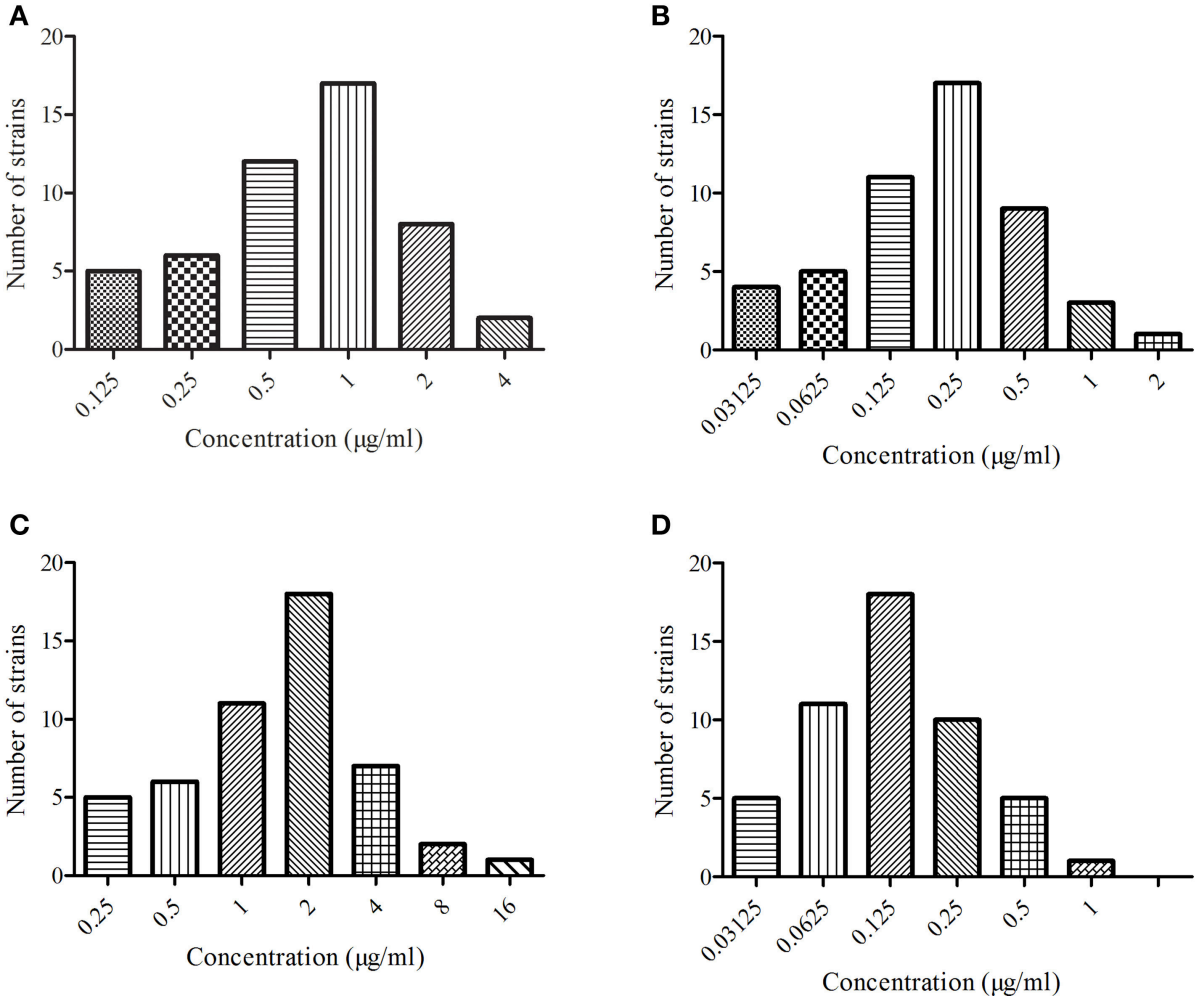
The MIC of marbofloxacin (Petsen) in four kinds of bacteria. **(A)** Represented MIC distribution of *Staphylococcus aureus*, **(B)** represented MIC distribution of *Escherichia coli*, **(C)** represented MIC distribution of *Pasteurella multocida*, **(D)** represented MIC distribution of *Streptococcus*.

The MIC distributions of MBF to the four kinds of bacteria are presented in Figure 1. The values of MIC ranged from 0.03 to 4.00 μg/ml, except for *E. coli*, which ranged from 0.25 to 16.00 μg/ml. The values of MIC_90_ of MBF against *S. aureus, E. coli, P. multocida*, and *Streptococcus* were 2.00, 4.00, 0.25, and 0.50 μg/ml, respectively. These findings indicated that these four kinds bacteria were sensitive to MBF, according to the CLSI M100-S23 guide document. Moreover, these results revealed that MBF had the excepted antimicrobial activity *in vitro*. MBF displayed a concentration-dependent killing action.

### Target animal safety evaluation

#### Clinical signs and mortality

**Figure 2:**
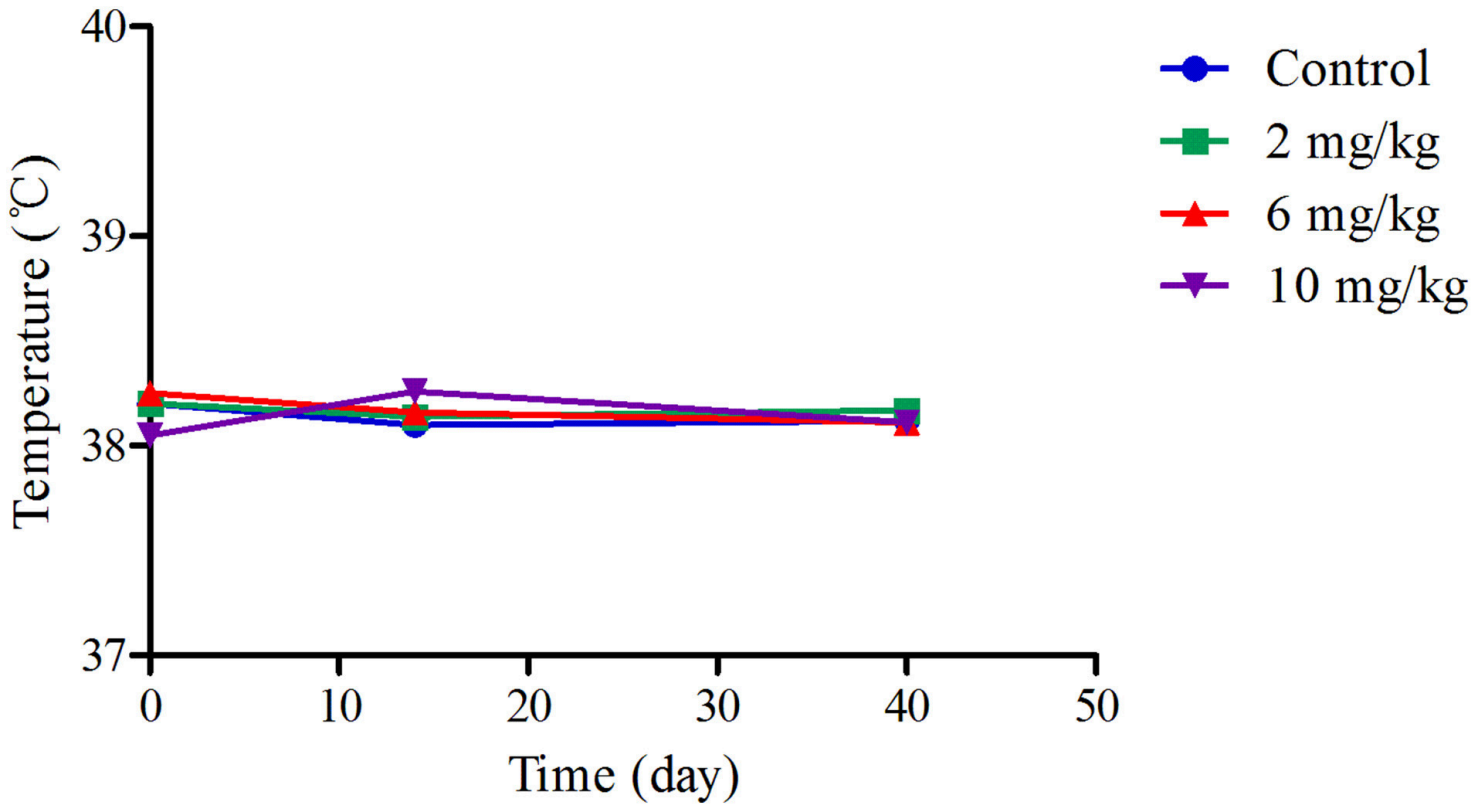
The mean of temperature and body weight in the 40 days feeding study. **(A)** Represented the mean of temperature.

In the MBF group, all the dogs survived, and no significant differences in coat condition, behavior, or mental condition were observed, in comparison with the control group. Body temperatures from the dogs in the low (2 mg/kg), middle (6 mg/kg), and high (10 mg/kg) dose groups were similar to each other, and ranged from 38.05 to 38.26°C; see Figure 2. Moreover, there were non-significant changes in feed consumption (14.72–16.54 kg) and body weight (8.96–10.44 kg) between the high, middle, low and dose groups and the control group (*p* > 0.05) at day 1, day 14, and day 40 of this study; see Supplemental Table 1.

#### Hematological examination

At day 1, 14, and 40, HGB, RBC, WBC, HCT, and PLT were tested; the results are presented in Table 1. There were no significant differences (*p* > 0.05) in these indicators between the low, middle, and high dose groups and the control group (*p* > 0.05). However, PLT was decreased and WBC was increased in the 6 and 10 mg/kg treatment groups (Table 1).

**Table 1:** Hematology parameters of beagle dogs on the day 0, 14, and 40 (Mean ± SD) after orally administration Petsen.

**Parameters**	**Control**			**2 mg/kg**			**6 mg/kg**			**10 mg/kg**		
	**Day 0**	**Day 14**	**Day 40**	**Day 0**	**Day 14**	**Day 40**	**Day 0**	**Day 14**	**Day 40**	**Day 0**	**Day 14**	**Day 40**
HGB (g/L)	163 ± 4.3	158.5 ± 4.6	162.8 ± 4.3	158 ± 4.1	159.2 ± 6.9	167.3 ± 4.9	162 ± 3.6	162.3 ± 5.2	168.2 ± 7.4	156 ± 9.9	155.3 ± 4.3	160.2 ± 5.2
RBC (10^12^/L)	6.9 ± 0.4	6.6 ± 0.1	6.9 ± 0.4	6.8 ± 0.2	6.6 ± 0.2	6.4 ± 0.2	6.9 ± 0.2	6.6 ± 0.1	6.9 ± 0.6	7.1 ± 0.4	6.4 ± 0.2	6.5 ± 0.4
WBC (10^9^/L)	10.9 ± 0.4	10.3 ± 0.2	10.2 ± 0.3	10.4 ± 0.4	10.2 ± 0.7	10.2 ± 0.3	10.8 ± 0.3	10.3 ± 0.2	10.8 ± 0.5	10.9 ± 0.5	10.9 ± 0.5	10.4 ± 0.4
HCT (%)	46.5 ± 2.4	44.5 ± 1.7	46.5 ± 2.4	46.0 ± 1.9	46.8 ± 3.6	47.4 ± 1.3	48.9 ± 1.3	47.4 ± 1.2	47.1 ± 1.8	48.3 ± 3.8	45.5 ± 4.1	47.5 ± 3.5
PLT (10^9^/L)	334 ± 25.7	337.5 ± 19.5	333.7 ± 25.7	361 ± 10.1	291.8 ± 59.2	343.8 ± 54.3	338 ± 33.8	293.7 ± 21.8	296.9 ± 35.5	330 ± 34.4	289.3 ± 45.4	292.7 ± 40.2

#### Serum biochemical analysis

The results of serum biochemical analysis were non-significant (*p* > 0.05) between the low, middle, high dose treatment groups, and control group excluding TBL, ALT, Na^+^, and AST which were significantly decreased, and ALP and BUN which were significantly raised (*P* < 0.05). These findings can be found in Table 2. However, these were not biologically significant changes and not biologically significant changes and these values did not fall outside the reference ranges.

**Table 2:** Serum biochemical analysis of beagle dogs on the day 0, 14, and 40 (Mean ± SD) after orally administration Petsen.

**Parameters**	**Control**			**2 mg/kg**			**6 mg/kg**			**10 mg/kg**		
	**Day 0**	**Day 14**	**Day 40**	**Day 0**	**Day14**	**Day 40**	**Day 0**	**Day 14**	**Day 40**	**Day0**	**Day 14**	**Day 40**
TC (mmol/L)	5.0 ± 0.1	5.0 ± 0.1	4.9 ± 0.1	4.6 ± 0.2	4.5 ± 0.1	4.7 ± 0.3	4.6 ± 0.3	4.3 ± 0.3	4.7 ± 0.2	47.3 ± 0.4	4.7 ± 0.5	4.9 ± 0.4
GLU (mmol/L)	4.5 ± 0.2	4.6 ± 0.2	4.5 ± 0.1	4.6 ± 0.3	4.4 ± 0.3	4.7 ± 0.2	4.6 ± 0.4	4.8 ± 0.4	4.4 ± 0.2	4.5 ± 0.3	4.8 ± 0.5	4.6 ± 0.3
Cr (μmol/L)	93.8 ± 0.9	93.5 ± 0.9	93.8 ± 0.6	92.9 ± 2.6	95.5 ± 6.0	94.5 ± 3.6	94.8 ± 3.7	96.5 ± 6.7	94.0 ± 2.8	93.9 ± 2.5	92.9 ± 3.9	93.5 ± 3.5
TBL (μmol/L)	1.2 ± 0.2	1.2 ± 0.2	1.1 ± 0.1	1 ± 0.1	0.6 ± 0.1^*^	0.4 ± 0.2^*^	1 ± 0.2	0.5 ± 0.1^*^	0.5 ± 0.2^*^	1.4 ± 0.2	0.6 ± 0.2^*^	0.5 ± 0.2^*^
ALT (U/L)	36 ± 2.0	35.9 ± 2.0	35.1 ± 2.1	36.3 ± 4.2	34.7 ± 3.1	31.8 ± 2.9^*^	35 ± 2.8	28.4 ± 3.5^*^	21.9 ± 1.6^*^	34.2 ± 2.7	30.9 ± 1.8^*^	22.7 ± 2.0^*^
AST (U/L)	23.2 ± 1.8	24.7 ± 2.6	24.5 ± 2.0	24.6 ± 2.9	23.6 ± 2.3	24.0 ± 2.7	21.7 ± 1.4	19.7 ± 3.1^*^	20.1 ± 1.9	23.7 ± 2.3	20.2 ± 1.4^*^	17.4 ± 2.3^*^
ALP (U/L)	92.8 ± 2.7	93.3 ± 2.4	92.7 ± 1.7	91.3 ± 3.4	91.5 ± 2.5	106 ± 3.1^*^	91.2 ± 3.0	109.9 ± 6.9^*^	105 ± 2.0^*^	106.4 ± 7.9^*^	115.4 ± 6.5^*^	115 ± 2.0^*^
TP (g/L)	59.2 ± 1.1	59 ± 0.8	58.7 ± 0.9	57.2 ± 0.8	58.9 ± 4.3	59.8 ± 5.7	57.6 ± 1.3	55.9 ± 1.9	55.6 ± 2.8	59.6 ± 0.8	54.4 ± 2.2	57.1 ± 0.9
ALB (g/L)	28.8 ± 0.8	29.4 ± 0.8	28.6 ± 0.6	28.6 ± 0.6	29.2 ± 1.6	31.1 ± 2.4	29.1 ± 0.5	31.2 ± 0.8	32.1 ± 1.3	28.1 ± 1.4	29.5 ± 1.1	30.3 ± 1.4
BUN (mmol/L)	3.3 ± 0.1	3.3 ± 0.1	3.3 ± 0.1	3.4 ± 0.3	3.1 ± 0.8	4.3 ± 0.8	3.3 ± 0.5	3.1 ± 0.8	4.1 ± 0.8	3.4 ± 0.3	3.4 ± 0.3	4.2 ± 0.6
K^+^ (mmol/L)	4.9 ± 0.1	4.9 ± 0.1	4.9 ± 0.1	5.0 ± 0.10	4.7 ± 0.1	5.0 ± 0.1	5.1 ± 0.2	5.1 ± 0.3	5.1 ± 0.2	5.1 ± 0.3	4.8 ± 0.1	5.0 ± 0.1
Na^+^ (mmol/L)	150.0 ± 1.6	147.5 ± 1.6	149.9 ± 1.4	148.4 ± 2.0	143.3 ± 2.7	149.6 ± 1.3	149.6 ± 1.1	143.8 ± 1.7^*^	150.5 ± 1.5	150.9 ± 1.9	143.5 ± 2.1^*^	150.8 ± 1.4
Cl^−^ (mmol/L)	102.7 ± 0.5	102.5 ± 0.8	102.8 ± 0.6	103.2 ± 1.0	103.1 ± 1.21	103.3 ± 1.1	103.2 ± 1.4	102.9 ± 0.6	103.6 ± 1.5	103.7 ± 0.8	102.1 ± 1.3	104.1 ± 0.7
Ca^++^ (mmol/L)	2.3 ± 0.07	2.3 ± 0.07	2.3 ± 0.1	2.4 ± 0.1	2.3 ± 0.2	2.4 ± 0.1	2.4 ± 0.1	2.2 ± 0.2	2.4 ± 0.1	2.3 ± 0.1	2.3 ± 0.2	2.35 ± 0.1

* *Present significant difference P < 0.05*.

#### Histopathological and organ examination

**Figure 3:**
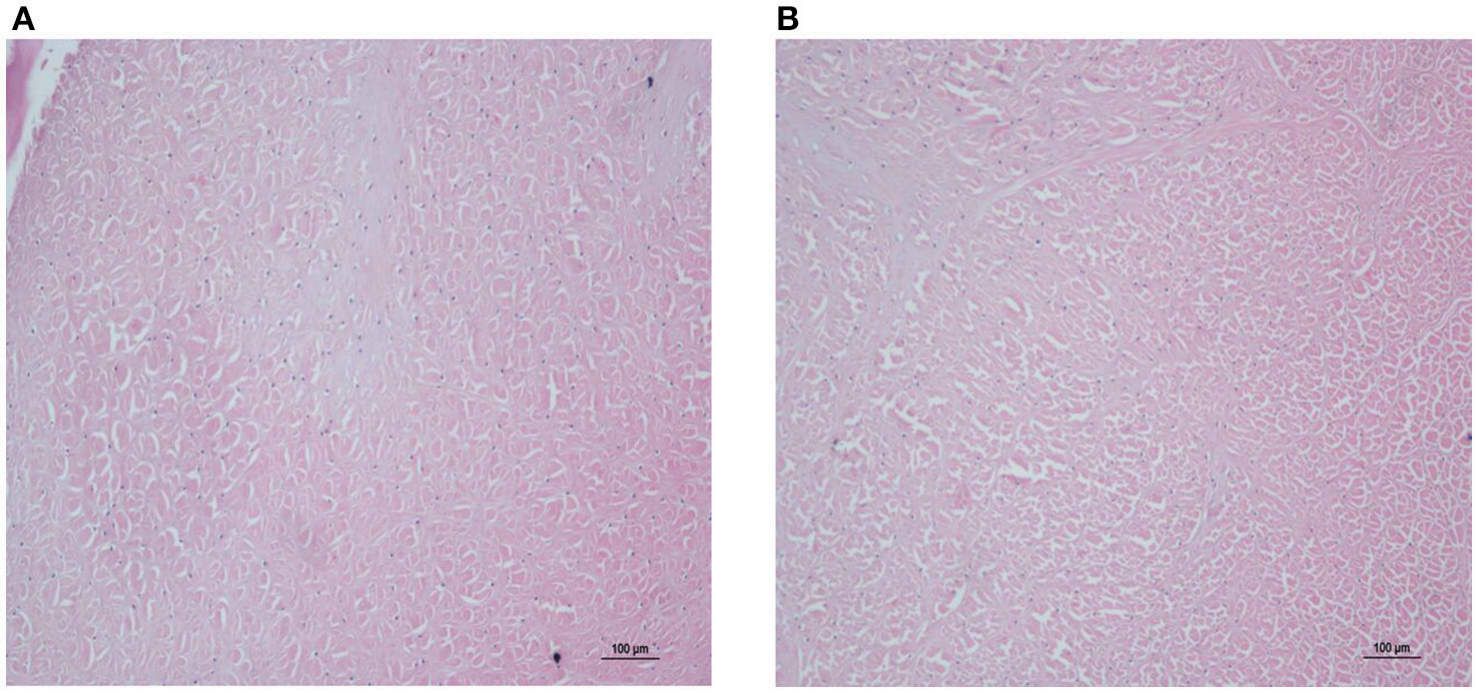
Microphotographs of articular cartilage in control and high dose treatment groups (10 mg/kg). **(A)** Represented control group, **(B)** represented high dose treatment group (10 mg/kg).

At 22 h after the last dose, the relative weights of the main organs (liver, heart, spleen, lungs, and kidneys) were calculated and are shown in Table 3. As compared to the control group, there were no significant differences in the low, middle, and high dose treatment groups. There were no histopathological findings in the organs examined. Articular cartilage from control dogs and dogs given 10 mg/kg MBF was investigated under microscopic examination; no differences were seen (see Figure 3).

**Table 3:** Relative weight of main organ in beagle dogs.

**Organs**	**Control (%)**	**2 mg/kg (%)**	**6 mg/kg (%)**	**10 mg/kg (%)**
Heart	0.894 ± 0.014	0.885 ± 0.026	0.890 ± 0.034	0.895 ± 0.016
Liver	3.055 ± 0.052	3.093 ± 0.071	3.124 ± 0.094	3.113 ± 0.081
Spleen	0.293 ± 0.021	0.286 ± 0.032	0.298 ± 0.044	0.296 ± 0.012
Lung	0.850 ± 0.029	0.837 ± 0.044	0.851 ± 0.036	0.841 ± 0.024
Kidney	0.503 ± 0.034	0.515 ± 0.019	0.512 ± 0.014	0.505 ± 0.022

### HPLC method validation

**Figure 4:**
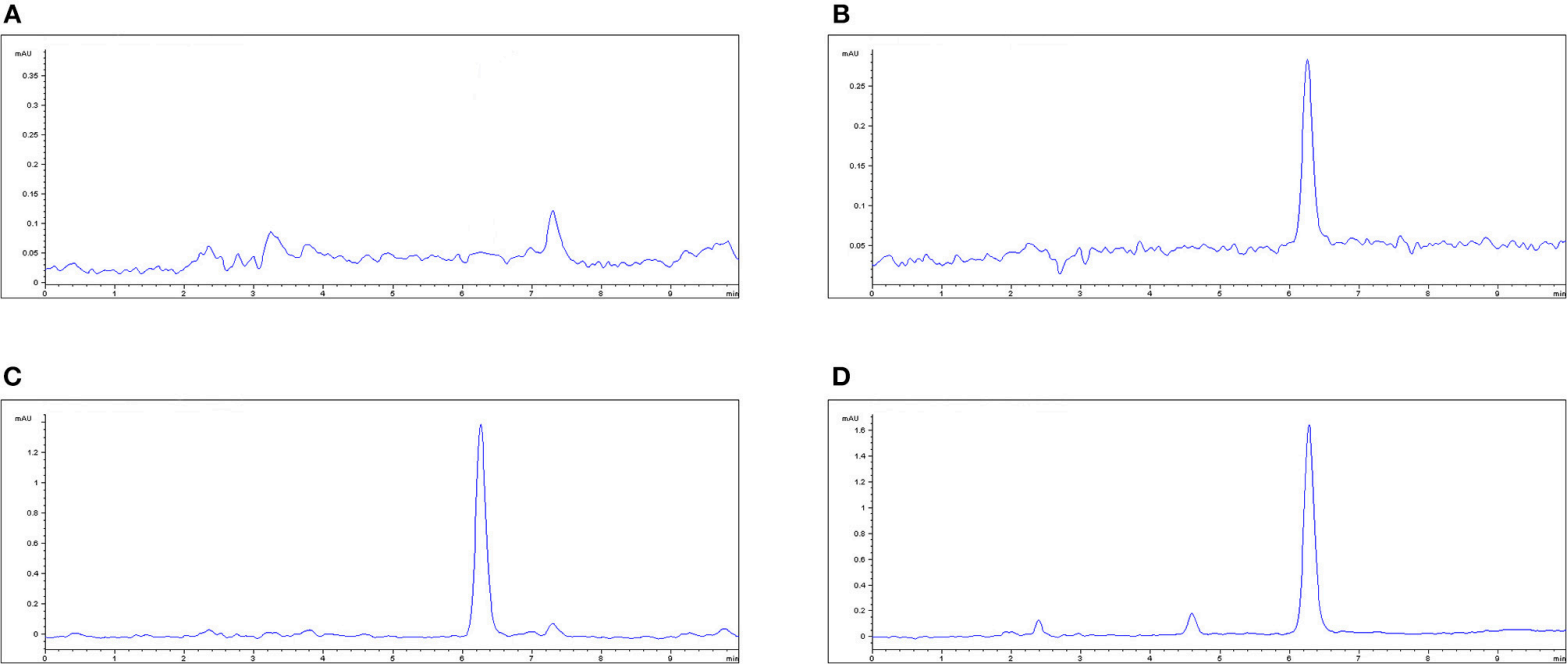
The HPLC method for MBF quantification in plasma. **(A)** Blank plasma sample, **(B)** plasma sample at the LLOQ of 0.05 μg/ml, **(C)** plasma sample after oral administration of Petsen at the point of 16 h, **(D)** plasma sample after i.v. administration of MBF at the point of 16 h. MBF at the peak time of 6.3 min.

The plasma limit of detection (LLOD) and limit of quantitation (LLOQ) of MBF was 0.02 and 0.05 μg/mL, respectively; see Figure 4. The proposed method of HPLC was suitable for MBF quantification in plasma. The recovery of MBF in plasma samples was higher than 85%. The intra-assay coefficients of variation for 0.02, 0.05, 0.50, and 5.00 μg/mL were <4.54%, and the inter-assay coefficients of variation for 0.02, 0.05, 0.50, and 5.00 μg/ml were 3.29, 2.07, and 1.33%, respectively. The typical regression equation was *y* = 40.737*x* – 2.2772, *R*^2^ = 0.996. The chromatogram is shown in Figures 4A–C; the blank is shown in Figure 4A, the lower limit of quantification (LLOQ) is shown in Figure 4B, and the measured plasma samples 16 h after oral and i.v. administration are shown in Figures 4C,D.

### Pharmacokinetics analysis

**Figure 5:**
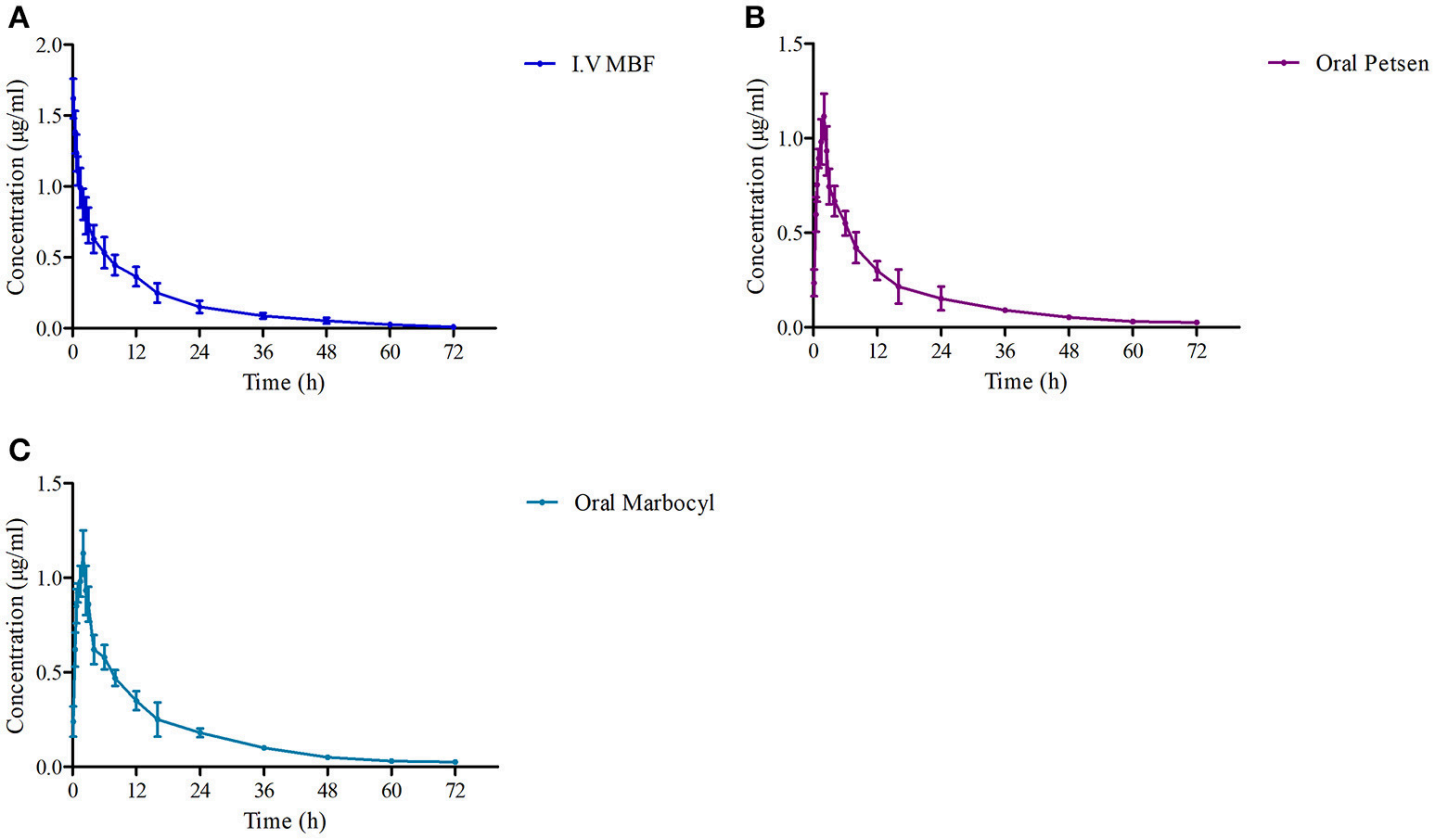
The curves of MBF concentration-time in plasma at a dose of 2 mg/kg after i.v. administrated **(A)** MBF, **(B)** oral Petsen, and **(C)** Marbocyl, respectively. MBF in plasma was determined at 0.17, 0.5, 0.75, 1, 2, 2.5, 3, 4, 6, 8, 12, 16, 24, 36, 48, 60, and 72 h.

The theoretical compartmental theoretical concentration-time profiles by non-linear regression equation analysis of MBF concentration-time profiles after oral Petsen, Marbocyl and i.v. MBF administrations are presented in Figure 5. After orally administrated Petsen, the theoretical compartmental theoretical concentration-time profiles of plasma were analyzed in accordance with an absorbing two-compartment open model; after i.v. administrated MBF, the concentration-time profile of plasma was analyzed in accordance with the non-compartment analysis. The main PK parameters of these two administration methods are shown in Table 4; these parameters were determined with WinNonlin software. The main parameters *t*_1/2_ or *t*_1/2β_, Cl_b_, AUC_0−∞_, *C*_max_, and *K*_*e*_ were 13.78 h, 0.14 L/h, 13.69 μg.h/ml, unavailable value and 0.053 h^−1^ after intravenous administrated MBF, 22.14 h, 0.15 L/h, 13.27 μg.h/ml, 0.95 μg/ml, and 0.09 h^−1^ after orally administrated Petsen, and 16.47 h, 0.14 L/h, 14.10 μg.h/ml, 0.97 μg/ml, and 0.11 h^−1^ after orally administrated Marbocyl. Moreover, the bioavailability values of Petsen and Marbocyl after oral administration were 97.11 and 101.70%, respectively.

**Table 4:** Main PK parameters after oral and i.v. administration in beagle dogs (*n* = 12).

**Parameters**	**Mean** ± **SD (*****n*** = **12)**		
	**MBF (i.v)**	**Petsen (orally)**	**Marbocyl (orally)**
*K_*e*_* (h^−1^)	0.053 ± 0.004	0.09 ± 0.01	0.11 ± 0.05
*t*_1/2_(h)	13.78 ± 1.21	–	–
*t*_1/2β_ (h)	–	22.14 ± 2.41	16.47 ± 2.18
Cl_b_ (L/h)	0.14 ± 0.08	0.15 ± 0.09	0.14 ± 0.04
MRT (h)	13.70 ± 1.48	21.73 ± 1.88	21.41 ± 3.36
AUC_0−∞_ (μg.h/ml)	13.69 ± 1.31	13.27 ± 1.48	14.10 ± 2.18
*T*_max_ (h)	–	1.46 ± 0.12	1.10 ± 0.38
*C*_max_ (μg/ml)	–	0.95 ± 0.14	0.97 ± 0.18
*F* (%)	–	97.11 ± 4.87	101.70%±5.12

*K_e_, elimination rate constant; t_1/2_ and t_1/2β_, half-life of elimination under non-compartmental and compartmental models; Cl_b_, total body clearance; MRT, mean residence time; AUC_0−∞_, area under curve from 0 to ∞; T_max_, time to the concentration peak; C_max_, the concentration in the peak; F, bioavailability*.

### Bioequivalence analysis

The mean ± SD of MBF concentrations-time profiles are presented in Figure 5 after oral two formulations, and the main descriptive PK parameters, are reported in Table 5.

**Table 5:** Pharmacokinetic parameters for the Test Formulation (Petsen) and Reference Formulation (Marbocyl), *p*-value, and relative fraction.

**Parameters**	**Unit**	**Petsen**	**Marbocyl**	**ANOVA**	***F* (%)**
AUC_0−∞_	μg.h/mL	13.27 ± 1.48	14.10 ± 2.18	0.313	94.11 ± 10.28
*C*_max_	μg/mL	0.95 ± 0.14	0.97 ± 0.18	0.874	
*T*_max_	H	1.46 ± 0.12	1.10 ± 0.38	>0.05^*^	

*F, represent relative bioavailability*.

* *Wilcoxon test*.

### Bioequivalence analysis

Log-transformed *C*_max_, AUC_0−∞_, and untransformed *T*_max_ of the test formulation (Petsen) were compared with the reference one (Marbocyl) for a bioavailability study with ANOVA analysis and 90% CI. It showed a significant difference in that *T*_max_ of Petsen (1.46 h) was longer than Marbocyl (1.10 h) in Table 5. This point might indicate the bioequivalence between Petsen and Marbocyl was not the same. This might be caused by a small magnitude and biological difference. However, no statistically significative differences were observed for *C*_max_ or AUC_0−∞_ in Table 5. The relative bioavailability of the test product compared to the reference one was 94.11 ± 10.28% (Table 5).

The two one-sided *T* tests estimated the ratios mean of log-transformed *C*_max_, AUC_0−∞_, and 90% CI on the test to reference formulations. Obtained values were 99.3, 99.2, and 91.9–107.2%, 92.0–102.1%, all in the range of 80–125% within the bioequivalence acceptance range (Table 6). These results demonstrated that Petsen was bioequivalent to the reference product (Marbocyl) in dogs.

**Table 6:** Two-one sided *T*-test and 90% confidence interval.

**Parameters**	**Petsen**	**Marbocyl**	**90% CI**	**Ratio (T/R) (%)**	**Acceptable range (%)**
AUC_0−∞_	13.27 ± 1.48	14.10 ± 2.18	92.0–102.1	99.2	80–125
*C*_max_	0.95 ± 0.14	0.97 ± 0.18	91.9–107.2	99.3	80–125

^*^Present significant difference P < 0.05.

## Discussion

Compared with the previously published reports and the PK profiles of Marbocyl, Petsen has also several advantages, including long-action, sustained release, and convenient administration to pets (Yang and Hu, 2006; Ghimire et al., 2007; Walther et al., 2014). In the present study, we performed a comprehensive toxicological evaluation of Petsen by conducting animal safety studies in beagle dogs. At the doses tested, Petsen was shown to be safe. In addition, this study also revealed the antibacterial activity of MBF from Petsen against four kinds of common pathogenic bacteria *in vitro*, as well as pharmacokinetic characteristics of MBF after administration of Petsen tablets *in vivo*.

In the safety study, no Petsen-related effects were observed in beagle dogs administered Petsen, in terms of mortality, morbidity, organ weight, body weight, total autopsy results, or microscopic manifestations in organ and histopathological examination. There were minimal differences in weight gain and food consumption among the control, low and middle dose groups, but an effect was seen at the highest dose used, as shown in Figure 2 and Supplemental Table 1. Moreover, there were also no treatment-related lesions based on the histopathology and examination of organs, as seen in Figure 3. It is known that administration of fluoroquinolones in animals and humans can cause toxicity such as gastrointestinal disturbances, anaphylaxis, hepatic and renal function injury, and in particular, articular cartilage lesions (Ball, 2000; Robinson et al., 2005; Thompson, 2007). Our study found no lesions in the articular cartilage among the three Petsen treatment groups (Supplemental Table 2). PLT was decreased and WBC was increased in the 6 and 10 mg/kg treatment groups on day 14 and 40 (Table 1). Further TBL, ALT, and AST were slightly decreased while ALP and BUN were slightly increased in the 6 and 10 mg/kg treatment groups, compared with the control group (Table 2). Moreover, there seemed to be a fall in Na across all groups at day 14th day compared to the control group. Although these indices presented significant differences in the test group compared to the control group on the 14 and 40th day (*P* < 0.05), these were not biologically significant changes; these values did not fall outside the reference ranges. Therefore, the 10 mg/kg b.w. the dose was regarded as the no observed adverse effect level (NOAEL) of Petsen in the current study. In a previous 13-week repeat-dose study, beagle dogs were given daily oral doses of 1, 4, and 40 mg/kg b.w. MBF in gelatin capsules. The typical quinolone-induced changes were observed at 40 mg/kg b.w. in the articular cartilage, and other toxic symptoms such as testicular tubular atrophy and spermatic granuloma also occurred in one dog at this dose. The recommended NOAEL was 4 mg/kg b.w. (Committee for Veterinary Medicinal Products, 2009a). Another study reported that no substance-related effects were found in immature dogs after being given doses of up to 6 mg/kg b.w. for 13 weeks, and the recommended NOAEL of MBF was 6 mg/kg b.w. (Committee for Veterinary Medicinal Products, 2009b). Moreover, in a two-generation study of rats fed diets containing 10, 70, and 500 mg/kg b.w., overt signs of toxicity such as impaired male fertility, reductions in implantation rate, litter size, and pup weight, as well as increased pup mortality were observed at doses of 10 and 500 mg/kg b.w. Therefore, the recommended NOAEL in rats was 10 mg/kg b.w. (Committee for Veterinary Medicinal Products, 2009b). In the present study, no significant toxicological effects were found up to 10 mg/kg in beagle dogs, and the NOAEL of Petsen was suggested to be 10 mg/kg. This Petsen dose is higher than the previously described report (4 mg/kg b.w.) in beagle dogs, but equal to the dose in rats. The difference for this might arises because of the decision to have dose of 4 and 40 mg/kg in the former study. This study provided a higher dose of NOAEL of MBF, which could be regarded as a reference in the future study.

Four kinds of bacteria with 50 strains were selected for MIC determination of Petsen. The MIC_90_ of these four kinds of bacteria (*S. aureus, E. coli, P. multocida, Streptococcus*) was 2.00, 4.00, 0.25, and 0.50 μg/ml, respectively (Figure 1). All of the MIC_90_ values were lower than 4 μg/ml, and the MIC_90_ of *P. multocida* and *Streptococcus* was lower than 1. *Streptococcus* was the most susceptible to Petsen. It had been reported that the MICs of MBF against the isolates of *E. coli, Streptococcus*, and *S. aureus*, isolated from pigs in China, were in the range of 0.13–0.25 μg/ml; other studies have also reported *E. coli* strains resistant to MBF whose MICs ranged from 8 to 32 μg/ml (Pellet et al., 2006; Ding et al., 2010; Andraud et al., 2011; Ferran et al., 2013). Our findings are similar to these previous reports, suggesting that these four kinds of bacteria are sensitive to Petsen, according to the CLSI M100-S23 guide document. MICs of three *E. coli* were up to 8 and 16 μg/ml. For the susceptibility breakpoint evaluation of *E. coli* to MBF, a previous study had suggested that MIC > 8 μg/ml was categorized as resistant (Andraud et al., 2011). However, based on the calculated MIC_90_ of *E. coli* to MBF (4 μg/ml), the MBF concentrations were focused on the intestinal tract, the site of infection with *E. coli*, and the *C*_max_ was 11.28 μg/ml in intestinal tract which was much higher than the MIC_90_ in the previously published report by Lei et al. (2017a). Therefore, our results suggest that Petsen will result in concentrations of MBF active against *E. coli*.

Following i.v. injection, the elimination half-life (*t*_1/2_) of MBF (13.78 h; as shown in Table 4) was much longer in beagle dogs than in broilers (5.26 h) and buzzards (4.11 h) (Garcia-Montijano et al., 2001; García-Montijano et al., 2003; Anadón et al., 2002; Haritova et al., 2006). Moreover, the value of *t*_1/2_ after i.v. administration of MBF was similar (13.78 h) to a previous report (Yohannes et al., 2015). However, after oral administration of Petsen at a dose of 2 mg/kg, the *t*_1/2β_ (22.14 h) value was higher than that in beagles (7.51 h) after intramuscular injection (i.m.) of MBF in the study by Yohannes (Yohannes et al., 2015), and also higher than that after i.v. administration in the current study. This difference was probably related to the continued absorption of MBF from the oral administration site in the period of the elimination phase, thereby prolonging the elimination phase time of MBF. Petsen showed high bioavailability, close to 100% (97.11%), after oral administration in beagle dogs (see Table 4). As the bactericidal activity of MBF was concentration-dependent, the high absorption and bioavailability could contribute to the bactericidal activity of MBF *in vivo*. The bioavailability of Petsen in beagle dogs in this study was comparable with other species, such as sheep, goats, and pigs, and was similar to that previously reported in beagle dogs; the bioavailability in all these animals has been shown to be close to 100% (Schneider et al., 1996; Waxman et al., 2001; Ding et al., 2010; Sidhu et al., 2010a,b; Marín et al., 2013). The high bioavailability may contribute to the prolonged elimination half-life after oral or i.m. administration; this may have induced a higher AUC. The *C*_max_ (1.10 μg/ml) of Petsen achieved in this study (Table 4) was higher than the MIC_90_ of *P. multocida* and *Streptococcus*, and was also higher than other breakpoints of fluoroquinolones recommended against susceptible bacteria, based on the CLSI M100-S19 guide document. The *C*_max_ (1.10 μg/ml) in this study was similar to that in pigs (1.03 μg/ml) (Ding et al., 2010; Marín et al., 2013). *C*_max_ obtained from orally administered MBF (1.10 μg/ml) was lower than that obtained from i.m. administration in pigs (1.81 μg/ml), as reported by Ding (Ding et al., 2010). Further, *C*_max_ in this study was lower than that reported by Yohannes (1.76 μg/ml) (Table 4).

For the bioequivalence trial of these two MBT preparations, and to perform a statistical comparison, AUC_0−∞_, *C*_max_, and *T*_max_ were chosen. When there are no statistically significant differences in these indices, bioequivalence is considered to have been shown (Vătăşescu et al., 2011; Marchidanu et al., 2013). In our findings, the three indices in Table 5 were revealed were non-significant between test (Petsen) and reference formulations (Marbocyl) (*p* < 0.05). The AUC_0−144h_ and AUC_0−∞_, *C*_max_ outcomes showing 90% of CI were inside the CIs (80–125%) set by the all guidelines. Therefore, these findings proved that the MBT-test product (Petsen) was bioequivalent to the reference one (Marbocyl).

As a tablet, oral administration of Petsen is recommended for pets. Thus, these results reveal that Petsen has high plasma concentration, wide distribution, and high bioavailability in beagle dogs, which supports its use as an alternative to Marbocyl.

## Conclusion

The results of this study revealed that, as a new formulation, Petsen has low toxicity in target animals (beagle dogs), antibacterial activity *in vitro*, and a pharmacokinetic profile in terms of high plasma concentration, wide distribution, long action, and bioequivalent which was similar to the reference one (Marbocyl). This study also provided a reasonable theoretical foundation for veterinary clinical application. Petsen might be conveniently and widely used for pets in veterinary clinic practice.

## Author contributions

JC: Conceived the study; QL and ZL: Designed the experiments. ZL, BF, and BY: Performed the experiments; ZL: Wrote the manuscript; QH, SA, HK, and JC: Improved the language. All authors reviewed the manuscript.

### Conflict of interest statement

The authors declare that the research was conducted in the absence of any commercial or financial relationships that could be construed as a potential conflict of interest.

## Abbreviations

MBF: Marbofloxacin (the active ingredient in marbofloxacin powder)
MBT: marbofloxacin tablet
PK: pharmacokinetics
EMA: European Medicines Agency
HPLC: high performance liquid chromatography
TSB: tryptic soy broth
TSA: tryptic soy agar
MIC: minimal inhibitory concentration
CLSI: Clinical and Laboratory Standards
OECD: Economic Cooperation and Development
FDA: Food and Drug Administration
RBC: red blood cell count
HGB: hemoglobin concentration
WBC: white blood cell count
HCT: hematocrit
PLT: blood platelet count; Urine analysis included ketone bodies
GLU: glucose
BIL: bilirubin
URO: urobilin
BLD: occult blood
PRO: protein
NIT: nitrite
ALP: alkaline phosphatase
AST: aspartate aminotransferase
ALT: alanine aminotransferase
ALB: albumin
TP: total protein
GLU: glutamate
BUN: blood urea nitrogen
CHOL: cholesterol
CREA: creatinine
CK: creatine kinase
TG: triglyceride
TBIL: total bilirubin
K: potassium
Na: sodium
Ca: calcium
CI: chloride
P: inorganic phosphorus
LLOD: the lower limit of detection
LLOQ: the lower limit of quantitation
*K*_*e*_: elimination rate of constant
*t*_1/2β_: half-life of elimination
Cl_b_: the total body clearance
MRT: mean residence time
AUC_0−∞_: area under curve from 0 to ∞
*T*_max_: time to the concentration peak
*C*_max_: the concentration in the peak
F: biovailability; Petsen (the test marbofloxacin tablet); Marbocyl (the reference marbofloxacin tablet).
