# Corrigendum: Evaluation of Marbofloxacin in Beagle Dogs After Oral Dosing: Preclinical Safety Evaluation and Comparative Pharmacokinetics of Two Different Tablets

**DOI:** 10.3389/fphar.2018.01385

**Published:** 2018-11-28

**Authors:** Zhixin Lei, Qianying Liu, Bing Yang, Haseeb Khaliq, Saeed Ahmed, Bowen Fan, Jiyue Cao, Qigai He

**Affiliations:** ^1^State Key Laboratory of Agriculture Microbiology, College of Veterinary Medicine, Huazhong Agriculture University, Wuhan, China; ^2^Department of Veterinary Pharmacology, College of Veterinary Medicine, Huazhong Agricultural University, Wuhan, China; ^3^National Reference Laboratory of Veterinary Drug Residues and MAO Key Laboratory for Detection of Veterinary Drug Residues, Huazhong Agriculture University, Wuhan, China

**Keywords:** fluoroquinolones, marbofloxacin, pharmacokinetics, beagle dogs, bioavailability, toxicity

In the original article, there was a mistake in Figure 4 as published. Information in Figure 4A was lost, due to a decimal error in the figure axis. The corrected Figure 4 appears below. The authors apologize for this error and state that this does not change the scientific conclusions of the article in any way. The original article has been updated.

**Figure 1:**
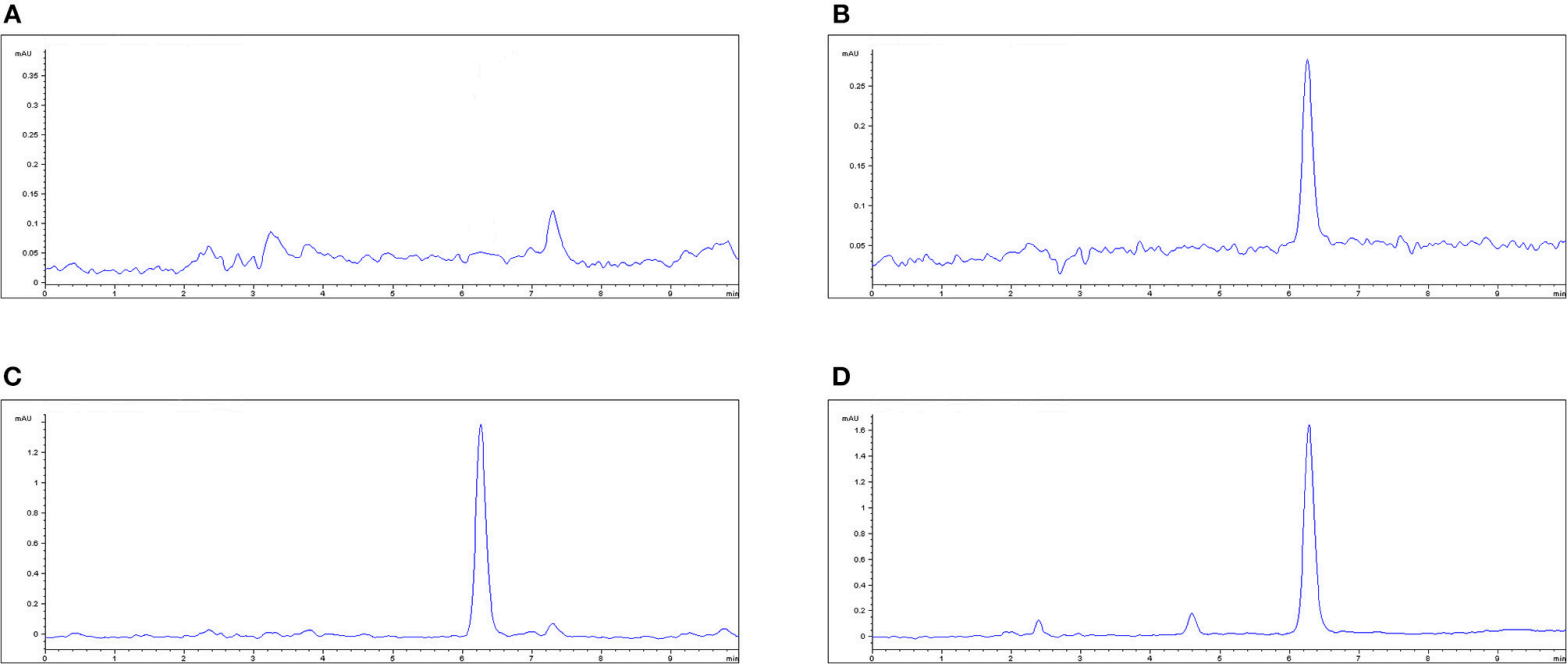
The HPLC method for MBF quantification in plasma. **(A)** Blank plasma sample, **(B)** plasma sample at the LLOQ of 0.05 μg/ml, **(C)** plasma sample after oral administration of Petsen at the point of 16 h, **(D)** plasma sample after i.v administration of MBF at the point of 16 h. MBF at the peak time of 6.3 min.

## Conflict of interest statement

The authors declare that the research was conducted in the absence of any commercial or financial relationships that could be construed as a potential conflict of interest.

