# Supplementary material for: Evaluation of Marbofloxacin in Beagle Dogs After Oral Dosing: Preclinical Safety Evaluation and Comparative Pharmacokinetics of Two Different Tablets
Source: Front Pharmacol. 2018 Apr 10;9:306. doi: 10.3389/fphar.2018.00306 (PMC5903334; doi:10.3389/fphar.2018.00306)
Supplement: Supplementary file 1 [file Table1.docx]

**Evaluation of Marbofloxacin** **in** **Beagle Dogs after oral dosing:** **Preclinical** **Safety Evaluation and Comparative Pharmacokinetics of Two Different Tablets**

**Zhixin Lei^abc^, Qianying Liu^abc^, Bing Yang^ab^, Haseeb Khaliq^b^, Saeed Ahmed^bc^, Bowen Fan^ab^, Jiyue Cao^bc*^, Qigai He^a*^**

^a^ State Key Laboratory of Agriculture Microbiology, College of Veterinary Medicine, Huazhong Agriculture University, Wuhan, China

^b^ Department of Veterinary Pharmacology, College of Veterinary Medicine, Huazhong Agricultural University, Wuhan, 430070, PR China

^c^ National Reference Laboratory of Veterinary Drug Residues and MAO Key Laboratory for Detection of Veterinary Drug Residues, Huazhong Agriculture University, Wuhan, 430070, PR China

***^*^Corresponding author:***

Prof. Ji-yue Cao, caojiyue2@163.com

Prof.Qi-gai He, he628@mail.hzau.edu.cn

**Table2. The mean** ± **standard deviation of weight gain and feed intake of beagle dogs**

| Groups (mg/kg) | 0^th^ day | 40^th^ day | Feed intake (kg) |
| --- | --- | --- | --- |
| Control | 9.34±0.50 | 10.44±0.45 | 15.32±1.98 |
| 2 | 9.03±0.78 | 10.42±0.44 | 15.64±2.23 |
| 6 | 9.51±0.75 | 10.38±0.49 | 14.72±1.67 |
| 10 | 8.96±0.45 | 10.13±0.39 | 16.54±2.56 |
