# Supplementary material for: Evaluation of Marbofloxacin in Beagle Dogs After Oral Dosing: Preclinical Safety Evaluation and Comparative Pharmacokinetics of Two Different Tablets
Source: Front Pharmacol. 2018 Apr 10;9:306. doi: 10.3389/fphar.2018.00306 (PMC5903334; doi:10.3389/fphar.2018.00306)
Supplement: Supplementary file 2 [file Table2.docx]

**Evaluation of Marbofloxacin** **in** **Beagle Dogs after oral dosing:** **Preclinical** **Safety Evaluation and Comparative Pharmacokinetics of Two Different Tablets**

**Zhixin Lei^abc^, Qianying Liu^abc^, Bing Yang^ab^, Haseeb Khaliq^b^, Saeed Ahmed^bc^, Bowen Fan^ab^, Jiyue Cao^bc*^, Qigai He^a*^**

^a^ State Key Laboratory of Agriculture Microbiology, College of Veterinary Medicine, Huazhong Agriculture University, Wuhan, China

^b^ Department of Veterinary Pharmacology, College of Veterinary Medicine, Huazhong Agricultural University, Wuhan, 430070, PR China

^c^ National Reference Laboratory of Veterinary Drug Residues and MAO Key Laboratory for Detection of Veterinary Drug Residues, Huazhong Agriculture University, Wuhan, 430070, PR China

***^*^Corresponding author:***

Prof. Ji-yue Cao, caojiyue2@163.com

Prof.Qi-gai He, he628@mail.hzau.edu.cn


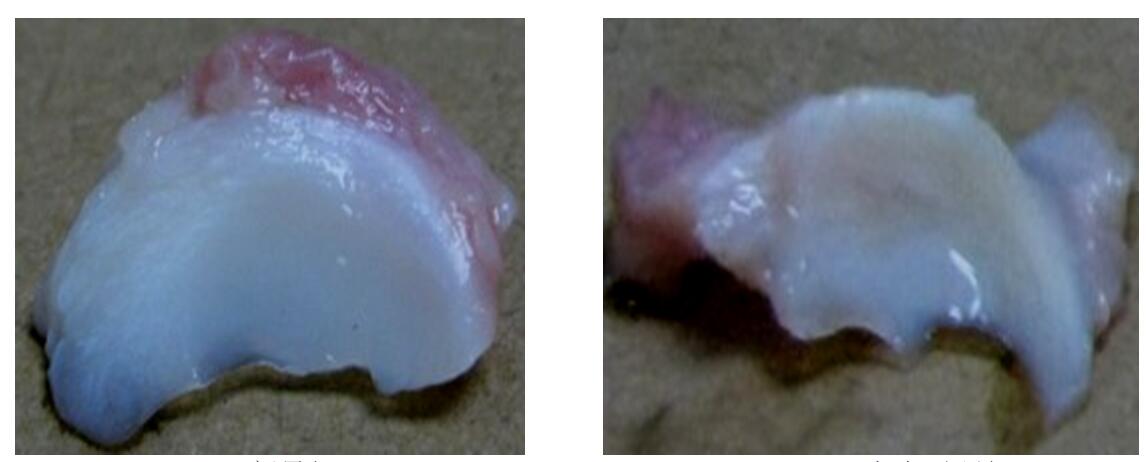


**Figure 1. Articular cartilage of beagle dogs in blank control group and the high dose group (10 mg/kg)**
